# Mesenchymal hepatic hamartoma: A rare case of severe respiratory distress in a neonate

**DOI:** 10.1002/ccr3.8562

**Published:** 2024-03-13

**Authors:** Kareem Omran, Jayasheela Kannan, Nisha Soares, Sameh Ali, Wissam Jamal Al Tamr

**Affiliations:** ^1^ Department of Public Health and Primary Care University of Cambridge Cambridge UK; ^2^ NMC Royal Hospital Sharjah United Arab Emirates

**Keywords:** gastroenterology and hepatology, general surgery, oncology, pediatrics and adolescent medicine, respiratory medicine

## Abstract

It is critical to consider intra‐abdominal pathology in cases of neonatal respiratory distress. Accurate and prompt diagnosis via computed tomography is lifesaving. We have highlighted the effectiveness of rapid surgical intervention as curative.

## Graphical abstract

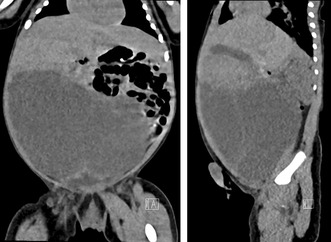

## 1 INTRODUCTION

Abdominal distension is a common presentation in neonates, with the most frequent etiology being congenital anomalies of the GI tract, such as intestinal atresia or malrotation.^1^ Although hepatic tumors are uncommon in the perinatal era, they can induce abdominal distension, be life‐threatening, and cause severe morbidity.^2^ Mesenchymal hamartomas of the liver most commonly occur in pediatrics and are the second most common benign liver tumors in children.^3^, ^4^ Traditionally, no unique clinical signs of this tumor have been observed, with the most prevalent signs being an abdominal mass or an enlarged liver.^5^ However, clinicians should be vigilant for symptoms such as an enlarged liver, a palpable abdominal mass, jaundice, and in some cases, related gastrointestinal or respiratory symptoms. Early recognition of these signs is crucial for prompt diagnosis and improved patient outcomes.

Radiological examination plays a pivotal role in the diagnosis of hepatic hamartomas. Typical findings on ultrasound, CT, or MRI may include a well‐defined mass with a mix of solid and cystic components. These imaging modalities aid in distinguishing hepatic hamartomas from other causes of neonatal abdominal distension.

In this report, we present a full‐term newborn with acute, severe abdominal distension of undefined etiology and worsening respiratory distress. Following full radiological examination and surgery, a large mesenchymal hepatic hamartoma was deemed the etiology. This condition's relevance in pediatric surgery was discussed, and the necessity for enhanced recognition of these tumors as surgical causes of infant respiratory distress is highlighted.

## 2 CASE HISTORY

A female newborn, delivered at 38 weeks with a birth weight of 3260 g, exhibited severe abdominal distension and signs of respiratory distress postdelivery. On physical examination, the newborn's abdomen was markedly distended, giving the abdomen a rounded appearance. On palpation, the abdomen was soft and nontender, with no distinct solid palpable mass. There were no signs of peritonitis. The liver edge was not palpable, and bowel sounds were present. APGAR scores were recorded as 8 and 9 at 1 and 5 min, respectively. Given the acute respiratory distress, the patient was admitted to the neonatal intensive care unit (NICU), where she was maintained in an incubator on room air and administered intravenous fluids.

## 3 INVESTIGATIONS

During the pregnancy, routine prenatal ultrasounds were performed. These were performed by the obstetrician, who noted no abnormalities, apart from potentially dilated bowel loops. The pregnancy was uneventful, with no known exposure to teratogens or significant maternal illness. The mode of delivery was a spontaneous vaginal delivery without complications. The family history was unremarkable with no known genetic disorders or similar cases of neonatal abdominal distension.

**FIGURE 1:**
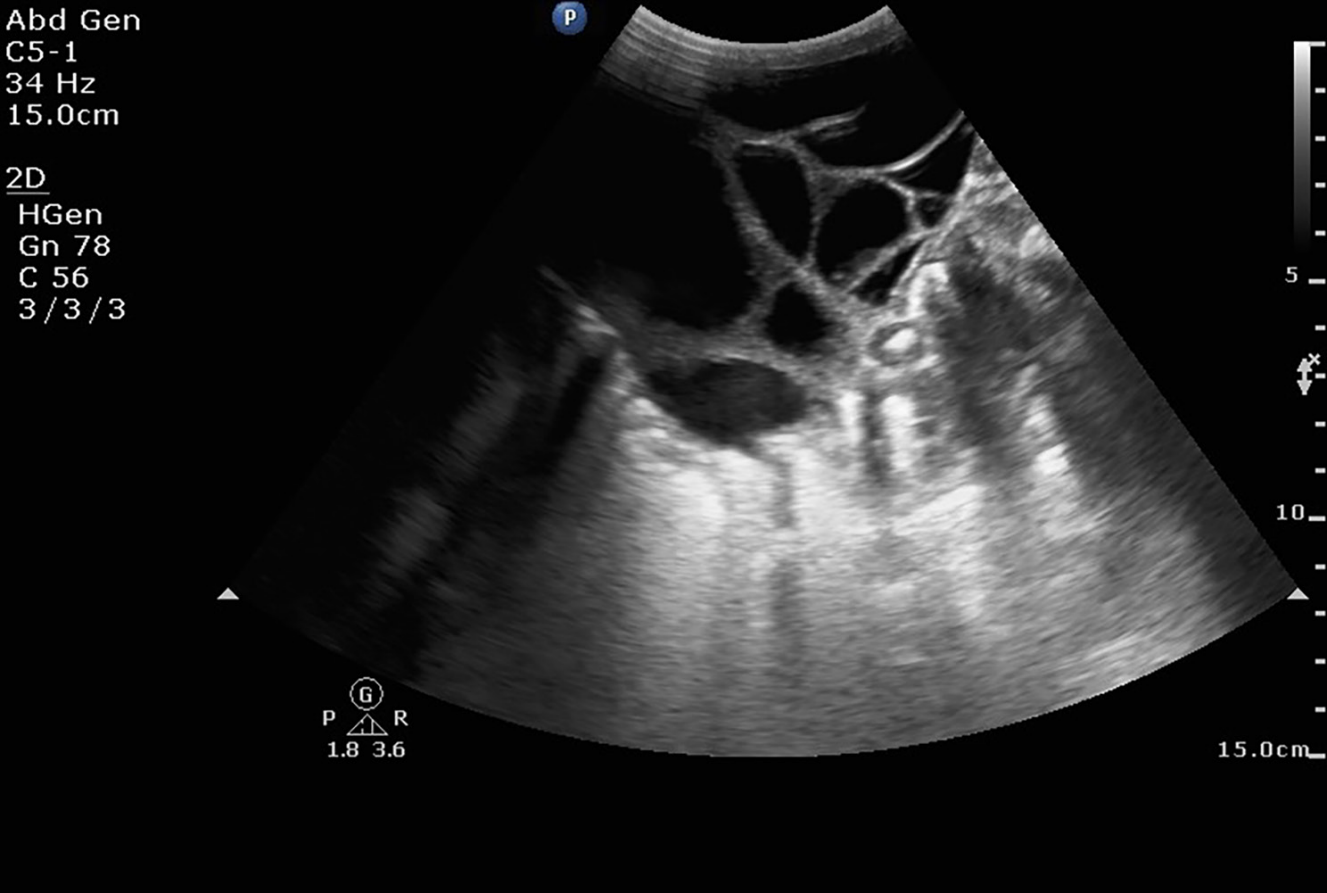
Presurgical abdominal ultrasound showing multiple cysts separated by setae was queried as multicystic dysplastic kidneys.

**FIGURE 2:**
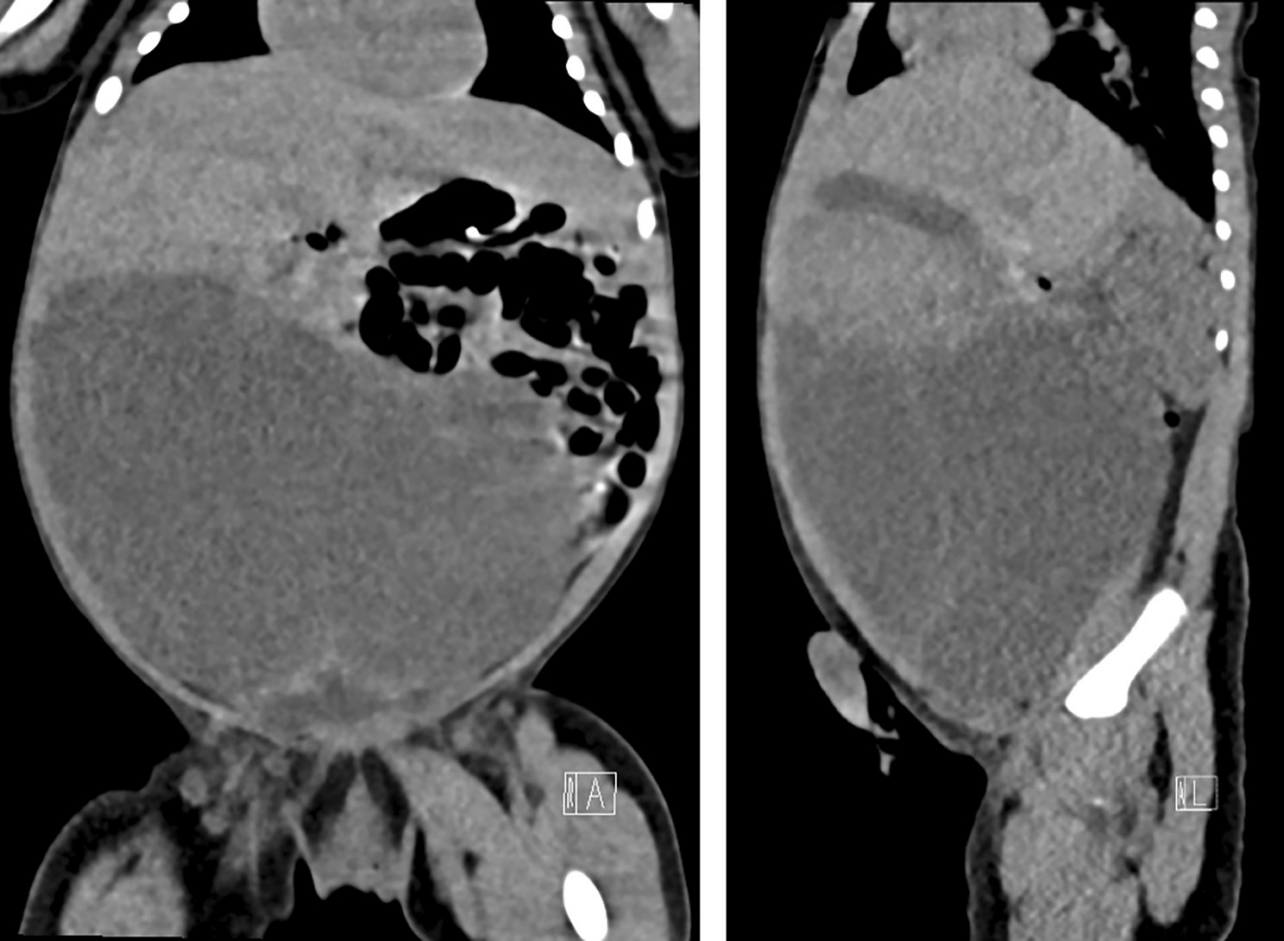
Coronal and sagittal computed tomography imaging showing a large cyst lesion of unknown origin, with a smooth outline. Thin septa are seen extending within the lesion. Attenuation values suggest serous fluid, being approximately +20 HU. No signs of hemorrhage or calcification seen.

Initial diagnostic efforts included an abdominal scan, which suggested the possibility of multicystic dysplastic kidneys (Figure 1). To further delineate the underlying pathology, a plain CT scan of the abdomen was performed. Contrary to the initial suspicion, the CT scan revealed a substantial cystic pelvi‐abdominal tumor, measuring approximately 10.6 cm × 7 cm × 8 cm, with thin septa extending within (Figure 2). Notably, both kidneys were visualized normally. The mass, located in the peritoneal compartment, caused significant displacement of bowel loops but showed no signs of hemorrhage, solid components, or calcification.

The decision to proceed with explorative laparotomy was influenced by several factors. The significant size of the mass and its impact on adjacent structures, combined with elevated alpha fetoprotein levels (66,422.9 ng/mL) and a normal beta HCG (17.6 mIU/mL), raised concerns for a neoplastic process. The presence of respiratory distress and acute severe abdominal distension in the patient further necessitated urgent surgical intervention for both diagnostic and therapeutic purposes.

## 4 TREATMENT

**FIGURE 3:**
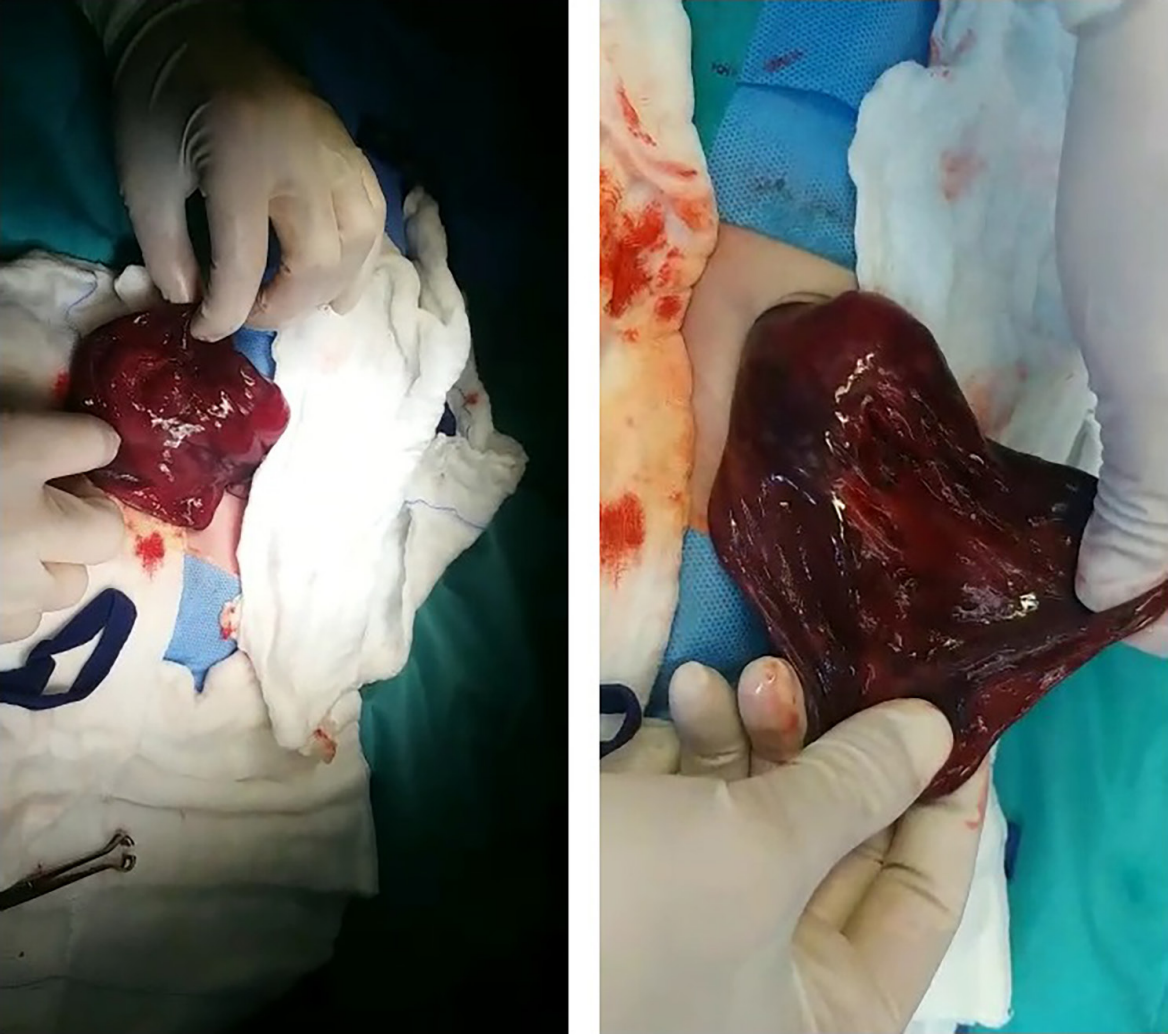
The large hepatic mass excised from the patient's right lobe of the liver. It measured greater than 15 cm in size and comprised of both solid and multi cystic components.

Due to the patient's escalating respiratory distress, an explorative laparotomy was performed on the second day of life. Surgery revealed a large hepatic mass (>15 cm) originating from the right lobe of the liver, characterized by solid and multicystic components (Figure 3). The mass was excised, necessitating partial hepatectomy of the right lobe and removal of the 6th and part of the 5th liver segments. The patient was then kept nil per mouth, and IV antibiotics were administered, escalated from vancomycin and Tazocin to meropenem and metronidazole due to elevated CRP levels and concerning aspirate.

## 5 OUTCOME AND FOLLOW‐UP

Postoperative management involved an MRI scan, primarily recommended by a pediatric oncologist. This step was taken to help rapidly characterize this mass whilst awaiting results from histopathology, which could take a few days in this hospital. The primary objectives of the MRI were to verify complete tumor excision, to investigate any additional components of the mass, and to assess for residual abnormal tissue, potential malignancy, or metastasis. Fortunately, the MRI results revealed no abnormalities, suggesting a successful surgical intervention free from immediate complications.

**FIGURE 4:**
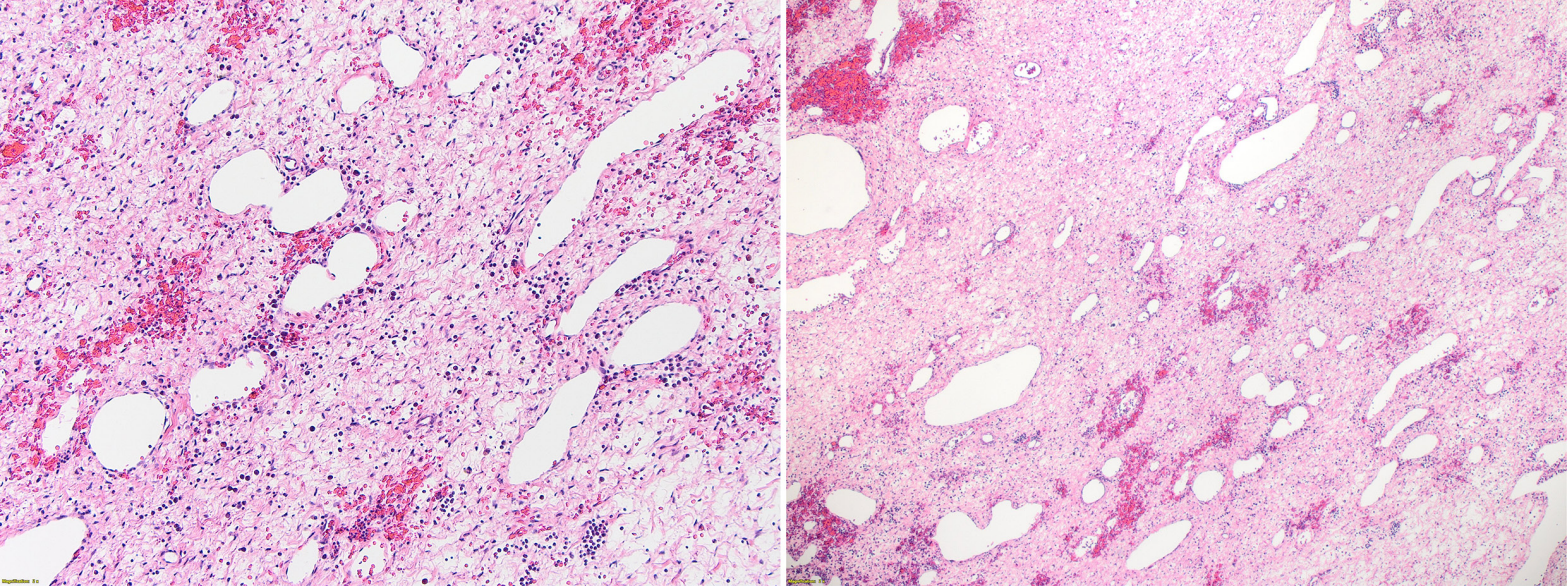
Histopathology sample showing myxomatous connective tissue containing loose mesenchymal tissue with interspersed branched and malformed bile ducts. Multiple cysts with no epithelial lining and patches of extramedullary hematopoiesis are noted.

The histopathological result arrived the following day, and examination of the excised tissue confirmed a diagnosis of a benign liver mass, a mesenchymal hamartoma. This showed myxomatous connective tissue containing loose mesenchymal tissue with interspersed branched and malformed bile ducts. Multiple cysts with no epithelial lining and patches of extramedullary hematopoiesis were noted. Fluid histopathology revealed no atypical or malignant cells (Figure 4).

Following normalization of CRP levels and negative cultures, antibiotic therapy was discontinued. Subsequent abdominal ultrasound revealed minimal subhepatic fluid. The patient was then discharged with no recurrence of symptoms observed 2 years postoperatively.

## 6 DISCUSSION

Hepatic mesenchymal hamartoma is a benign tumor composed of myxoid mesenchymal tissue with fluid accumulation.^6^ Since its initial characterization by Edmondson in 1956, HM has been reported with an incidence of approximately 0.7 cases per million population annually.^7^, ^8^ Despite its rarity, the clinical implications of HM are significant, particularly in neonates, where the tumor's rapid postnatal growth can lead to acute presentations.

The first line of imaging in suspected HM cases typically involves ultrasonography, which can variably present the tumor as multicystic, mixed solid‐cystic, or completely solid.^9^, ^10^ However, the operator‐dependent nature of ultrasonography can lead to diagnostic inaccuracies, as evidenced in our case where it erroneously suggested multicystic dysplastic kidneys. Consequently, computed tomography (CT) scans become crucial, offering a more definitive approach to localizing and characterizing these tumors, particularly in complex cases.

The clinical presentation of this tumor varies by age; however, neonates tend to have a more acute clinical presentation due to rapid growth after birth as a result of fluid accumulation in the cysts.^11^ If this growth is significant enough, most symptoms arise due to the mass effect caused on surrounding structures.^12^ In our case, the patient's cysts included a volume greater than 300 mL, which caused compression of the diaphragm and induced respiratory distress in this patient. While respiratory distress is not uncommon in neonates, affecting up to 7% of newborns, it is very rarely attributed to hepatic tumors.^13^ There have been cases where hepatic mesenchymal hamartomas were associated with respiratory distress in a neonate, including instances with malrotation and placental abnormalities.^14^, ^15^, ^16^ However, due to the scarcity of literature on this presentation, it is unknown whether these are incidental.

Although mesenchymal hamartomas are benign, their management is not always straightforward. Previous literature has suggested that conservative management may be appropriate in some cases.^17^ However, our experience emphasizes the need for prompt surgical intervention in instances where the tumor size is substantial and poses risks such as diaphragmatic compression.^18^ Further, a recent systematic review was in support of this approach, and concluded that complete tumor resection should remain the gold standard, owing to the comparatively low morbidity rates.^19^ In our case, the emergent surgical approach not only alleviated the immediate life‐threatening respiratory distress but also provided a definitive treatment for the mesenchymal hamartomas.

Given the rarity of mesenchymal hamartomas and their variable presentations, there is a need for heightened awareness among clinicians regarding this condition as a potential cause of respiratory distress in neonates. Our case highlights the importance of considering intra‐abdominal pathology in neonatal respiratory distress and the effectiveness of rapid, decisive surgical intervention. Additionally, further studies are warranted to explore the long‐term outcomes of patients undergoing surgery for HM in the neonatal period.

In conclusion, we present a case of a massive hepatic mesenchymal hamartoma independently resulting in respiratory distress in a neonate. The importance of this case is due to the acute and life‐threatening presentation, which has not yet been reported in the literature. Hence, we would like to inform and emphasize the need for prompt accurate diagnosis and management. It is essential to be aware of intra‐abdominal pathology and hepatic masses in cases of neonatal respiratory distress, as well as the requirement for CT to avoid misdiagnosis. We have placed emphasis on the successful nature of rapid surgical intervention in inducing resolution of symptoms and as curative management of the condition.

## AUTHOR CONTRIBUTIONS

**Kareem Omran:** Data curation; formal analysis; investigation; resources; validation; writing – original draft; writing – review and editing. **Jayasheela Kannan:** Investigation; methodology; supervision; writing – review and editing. **Nisha Soares:** Conceptualization; investigation; resources; supervision; writing – review and editing. **Sameh Ali:** Conceptualization; investigation; resources; supervision; writing – review and editing. **Wissam Jamal Al Tamr:** Conceptualization; investigation; methodology; project administration; supervision; writing – review and editing.

## CONFLICT OF INTEREST STATEMENT

We disclose no conflict of interest, any sources of support or external funding.

## CONSENT

Written informed consent was obtained from the patient to publish this report in accordance with the journal's patient consent policy.

## Data Availability

Data sharing not applicable to this article as no datasets were generated or analysed during the current study.
